# Comparison of three different kits for extraction of high-quality RNA from frozen blood

**DOI:** 10.1186/2193-1801-3-76

**Published:** 2014-02-08

**Authors:** Jin-Hee Kim, Hyeon-Ok Jin, Jin-Ah Park, Yoon Hwan Chang, Young Jun Hong, Jin Kyung Lee

**Affiliations:** KIRAMS Radiation Blood Specimen Biobank, Korea Institute of Radiological & Medical Sciences, 215-4 Gongneung-dong, Nowon-gu, Seoul, 139-709 Republic of Korea; Department of Laboratory Medicine, Korea Cancer Center Hospital, Korea Institute of Radiological & Medical Sciences, 215-4 Gongneung-dong, Nowon-gu, Seoul, 139-709 Republic of Korea

**Keywords:** Frozen blood, RNA extraction, RT-qPCR

## Abstract

Extraction of high-quality RNA is a crucial step in gene expression profiling. To achieve optimal RNA extraction from frozen blood, the performance of three RNA extraction kits- TRI reagent, PAXgene blood RNA system (PAXgene) and NucleoSpin RNA blood kit (NucleoSpin)- was evaluated. Fifteen blood specimens collected in tubes containing potassium ethylenediaminetetraacetic acid (EDTA) and stored at −80°C for approximately 5 years were randomly selected. The yield and purity of RNA, RIN (RNA integrity number) values and cycle threshold (Ct) values were assessed. Mean RNA yields with TRI reagent, PAXgene and NucleoSpin were 15.6 ± 8.7 μg/ml, 3.1 ± 1.7 μg/ml and 9.0 ± 5.5 μg/ml, respectively. Mean A_260_/_280_ ratios of RNA for the three kits were 1.7 ± 0.1, 2.0 ± 0.1, and 2.0 ± 0.0, and mean RIN values recorded as 3.2 ± 0.8, 6.0 ± 1.1, and 6.4 ± 0.9, respectively. The Ct values of housekeeping genes, *18S rRNA*, *β-actin*, *RPLP0* and *HPRT1*, were as follows: TRI reagent (19.2 ± 1.6, 30.6 ± 1.8, 29.9 ± 1.4 and 36.3 ± 1.3), PAXgene 16.6 ± 1.4, 26.4 ± 1.3, 28.2 ± 1.8 and 33.8 ± 1.1), and NucleoSpin (16.3 ± 1.5, 27.2 ± 1.3, 27.0 ± 1.6 and 32.9 ± 1.6). RNA yield using TRI reagent was 1.7 times higher than that with NucleoSpin and 5 times higher than that with PAXgene. However, the purity and integrity of TRI-extracted RNA was lower than that extracted with PAXgene and NucleoSpin. Moreover, the Ct values of housekeeping genes after extraction with TRI reagent were approximately 1.7-3.8 times higher than those obtained with PAXgene and NucleoSpin. The PAXgene and NucleoSpin kits produced similar results in terms of RNA purity and integrity and subsequent gene amplification. However, RNA yields from NucleoSpin were 2.9-fold higher, compared to PAXgene. Based on these findings, we conclude that NucleoSpin is the most effective kit for extraction of abundant and high-quality RNA from frozen blood.

## Background

Human blood specimens are routinely collected by biobanks for medical applications, scientific research, and diagnostic purposes (Elliott and Peakman 2008; Jackson and Banks 2010; Hebels et al. 2013). Blood specimens collected in tubes containing anticoagulants are usually processed to produce aliquots of whole blood, serum, plasma, or buffy coats, which are stored at −80°C for future use. Frozen blood samples stored in biobanks are increasingly considered valuable sources of RNA for profiling gene expression (Barnes et al. 2010). However, a major limitation of studies using RNA isolated from frozen blood samples is RNA degradation during blood collection and storage.

To minimize the degradation of RNA and changes in gene expression patterns, improved alternatives for collecting human blood specimens have been developed (Chai et al. 2005). For instance, PaxGene Blood RNA tubes (PAXgene, PreAanlytix, Hombrechtikon, Switzerland) containing RNA stabilizing reagents are used to collect and store blood samples prior to RNA isolation. Despite the availability of an RNA stabilization system, there may be a need to perform RNA expression profiling experiments with legacy blood samples initially collected using common blood collection tubes (e.g. EDTA tubes) (Chai et al. 2005; Thörn et al. 2005). Recently, Beekman and co-workers proposed an effective way of extracting RNA from frozen blood samples collected using EDTA tubes. Thawed blood is transferred to PAXgene Blood RNA tubes, and RNA obtained using the PAXgene RNA extraction kit. This transfer method yields abundant RNA of sufficient quality for gene expression analysis (Beekman et al. 2009).

The most common RNA extraction kits for frozen blood are based on the use of an acid guanidinium thiocyanate-phenol-chloroform extraction reagent. The TRI reagent (Invitrogen, Carlsbad, CA, USA) is based on this technology and rapidly inhibits RNase activity, making it a complete, ready-to-use reagent for total RNA extraction (Kang et al. 2011; Jakovljevic et al. 2010).

Another product, the NucleoSpin RNA Blood kit (NucleoSpin, MACHEREY-NAGEL, Duren, Germany), facilitates extraction of RNA from blood samples collected in EDTA blood collection tubes.

In the current study, three different RNA extraction kits, specifically, TRI reagent, PAXgene, and NucleoSpin, were compared in terms of RNA yield, purity, and integrity, with a view to achieving optimal high-quality RNA extraction from frozen whole blood. RNA extracted with the three kits was additionally assessed for use in real-time PCR assays.

## Results and discussion

**Figure 1:**
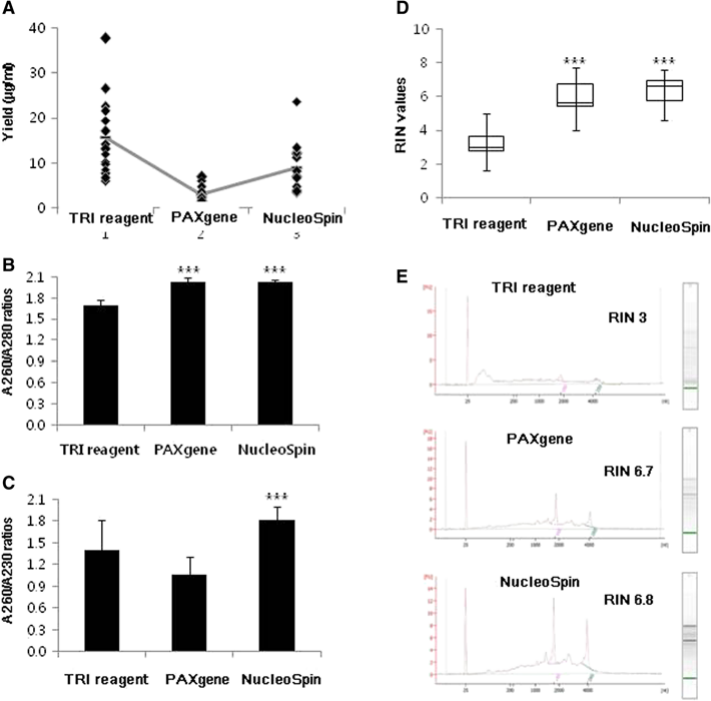
**Yield, purity, and integrity of RNA isolated from frozen blood using three different extraction kits. (A)** The graph shows individual yields (n = 15). **(B, C)** The graphs show mean A_260_/A_280_ and A_260_/A_230_ ratios. Bars indicate standard deviations. **(D)** The graph depicts a box-and-whisker plot of variations in RIN values. The upper and lower edges of the boxes represent the 25% and 75% lines, and the central line shows median values. **(E)** RNA was run on a eukaryote total RNA Nano chip. 18S and 28S peaks of sample #2 are shown. ***p < 0.001 versus TRI reagent (b). ***p < 0.001 versus TRI reagent or PAXgene **(C)**.

The mean yields of RNA isolated from 15 frozen whole blood samples with the three different kits were as follows: TRI reagent, 15.6 ± 8.7 μg/ml (range, 6.1 ~ 37.6), PAXgene, 3.1 ± 1.7 μg/ml (range, 1.1 ~ 6.9), and NucleoSpin, 9.0 ± 5.5 (range, 3.6 ~ 23.7) μg/ml (Table 1 and Figure 1A). TRI reagent yielded the highest quantity, generally resulting in extraction of 5.0-fold more RNA than the PAXgene kit and 1.7-fold more RNA than the NucleoSpin kit. However, RNA extracted with TRI reagent exhibited lower purity than that obtained using either PAXgene or NucleoSpin (Table 1 and Figure 1B). RNA absorbs UV light maximally at 260 nm and protein at 280 nm, and A_260_/A_280_ ratios are thus used to indicate RNA sample purity. The mean RNA purity (A_260_/A_280_) was evaluated as 1.7 ± 0.1 with TRI reagent, 2.0 ± 0.1 with PAXgene, and 2.0 ± 0.0 with NucleoSpin. Generally, samples with ratios in the range of 1.8 ~ 2.0 indicate higher levels of RNA purity. The A_260_/A_280_ ratios for PAXgene and NucleoSpin extractions were within the desired range, while the ratio for TRI reagent was somewhat lower, indicative of protein or DNA contamination with this kit. As a secondary measurement of RNA purity, A_260_/A_230_ ratios were additionally evaluated for extracts with TRI reagent (1.4 ± 0.4), PAXgene (1.1 ± 0.3), and NucleoSpin (1.8 ± 0.2) (Table 1 and Figure 1C). The mean A_260_/A_230_ ratios for RNA obtained with PAXgene and TRI reagent were lower than that with Nucleospin. Phenol, carbohydrate and salt contamination are reported to induce a decrease in the A_260_/A_230_ ratio. In this regard, the lower A_260_/A_230_ values may be attributed to the high salt content of PAXgene elution buffer and phenol in TRI reagent.

**Table 1:** **Yield, purity, and integrity of total RNA isolated from frozen blood using three different extraction kits: TRI reagent, PAXgene, and NucleoSpin (n = 15)**

	RNA yield (μg/ml)	RNA purity (A260/A280) (A260/A230)	RNA integrity (RIN)
**TRI reagent**	15.6 ± 8.7 (6.1-37.6)	1.7 ± 0.1 (1.6-1.7) 1.4 ± 0.4 (0.7-2.1)	3.2 ± 0.8 (1.6-5.0)
**PAXgene**	3.1 ± 1.7 (1.1-6.9)	2.0 ± 0.1 (1.9-2.1) 1.1 ± 0.3 (0.6-1.5)	6.0 ± 1.1 (4.0-7.7)
**NucleoSpin**	9.0 ± 5.5 (3.6-23.7)	2.0 ± 0.0 (2.0-2.1) 1.8 ± 0.2 (1.5-2.1)	6.4 ± 0.9 (4.6-7.6)

Data are presented as means ± standard deviation. Numbers in parentheses represent the data range.

RNA integrity was assessed using an Agilent 2100 BioAnalyzer, which provides RIN scores for RNA quality control. The mean RIN values were 3.2 ± 0.8 for TRI reagent, 6.0 ± 1.1 for PAXgene, and 6.4 ± 0.9 for NucleoSpin (Table 1 and Figure 1D), signifying that the PAXgene and NucleoSpin kits yield RNA with better integrity than the TRI reagent.

**Figure 2:**
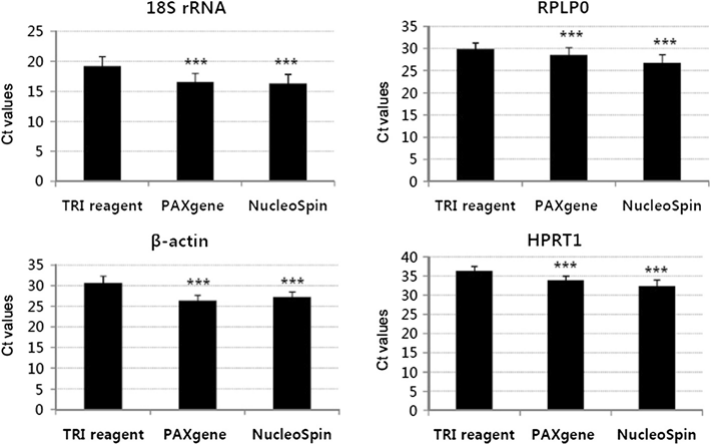
**Graph of cycle thresholds (Ct) of three housekeeping genes assessed for RNA samples extracted using three different kits (TRI reagent, PAXgene, and NucleoSpin) and amplified using real time RT-PCR (n = 15).** Bars indicate standard deviations. ***p < 0.001 versus TRI reagent.

To further validate the quality of RNA obtained using the three different extraction kits, real-time PCR analysis was performed. The expression patterns of four housekeeping genes, *18S rRNA*, *β-actin*, *RPLP0* and *HPRT1*, were analyzed and presented as cycle threshold (Ct) values. As shown in Figure 2, the Ct values obtained from TRI reagent, PAXgene and NucleoSpin extractions were 19.2 ± 1.6, 16.6 ± 1.4 and 16.3 ± 1.5 for *18S rRNA*, 30.6 ± 1.8, 26.4 ± 1.3 and 27.2 ± 1.3 for *β-actin*, 29.9 ± 1.4, 28.2 ± 1.8 and 27.0 ± 1.6 for *RPLP0*, and 36.3 ± 1.3, 33.8 ± 1.1 and 32.9 ± 1.6 for *HPRT1*, respectively. The Ct values for RNA extracted using TRI reagent were ~1.7 to 3.8 times higher than those evaluated with the PAXgene and NucleoSpin kits, clearly indicating that RNA extracted with TRI reagent is of lower quality.

Among the three kits, TRI reagent produced the highest RNA yield. However, the purity of TRI-extracted RNA (A_260_/_280_ ratio of 1.7) and integrity (RIN 3.2) were lower, compared to RNA extracted using the PAXgene (A_260_/_280_ ratio 2.0, RIN 6.0) and NucleoSpin (A_260_/_280_ ratio 2.0, RIN 6.4) systems. Earlier, Fleige and co-workers reported that RNA with low RIN values shows reduced PCR performance due to the presence of more highly fragmented molecules, leading to inaccurate gene expression evaluation (Fleige and Pfaffl 2006 Fleige et al. 2006). Gene expression levels of *c-fos*, *IL-1β*, *IL-8*, and *GAPDH* were decreased in blood samples with RIN values below 5 (Pazzagli et al. 2013). RIN values of the RNA extracted with PAXgene and NucleoSpin were higher than the cut-off value of 5, suggesting that RNA extracted using these kits is of similar quality and performs well in standard gene expression analysis (Beekman et al. 2009; Pazzagli et al. 2013). In the current study, data obtained with real-time PCR revealed lower Ct values of the housekeeping genes, *18S rRNA*, *β-actin*, *RPLP0* and *HPRT1*, measured from PAXgene and NucleoSpin extractions, compared to those measured from TRI reagent-extracted RNA (Figure 2). RNA extracted with TRI reagent can contain trace genomic DNA contamination, which presents significant problems, especially for PCR-based applications (Jiang et al. 2013). In our experiments, RNA isolated using TRI reagent was not treated with DNase I. Thus, we propose that TRI reagent-extracted RNA is contaminated with genomic DNA, thereby showing high RNA yield that is overestimated, low in purity and poorly amplified.

The increased availability of blood collection systems with RNA-stabilizing additives and accompanying RNA extraction kits has significantly improved the quality of RNA isolated from blood (Rainen et al. 2002; Thach et al. 2003). However, there remains a need to assess RNA quality and profiling experiments in blood specimens collected with common blood collection systems (EDTA tubes). Beekman and co-workers proposed a transfer method of thawed EDTA blood into the PAXgene system, which is efficient in terms of yield and quality of RNA, compared to the standard protocol (QIAamp RNA blood kit, Qiagen, Valencia, CA, USA) (Beekman et al. 2009). However, this method could not be expected to reverse gene expression changes and variability or RNA quality loss that may have occurred after blood samples originally collected without RNA-stabilizing additives (Beekman et al. 2009).

The PAXgene and NucleoSpin kits produced similar results in terms of RNA purity and integrity and subsequent gene amplification. Our results indicate that RNA extracted from frozen blood samples using the PAXgene and NucleoSpin kits is of high quality, which should facilitate gene expression analyses, such as the real-time PCR assay. However, the RNA yield from NucleoSpin was higher than that from PAXgene. Based on the collective findings, we conclude that extraction using the NucleoSpin kit is the most effective way to produce abundant and high-quality RNA from frozen blood.

## Materials and methods

### Samples and RNA extraction procedure

Blood specimens were collected with informed consent from cancer patients undergoing radiation treatment in Korea Cancer Center Hospital. Blood was collected into potassium EDTA tubes (Greiner Bio-One, Frickenhausen, Germany), transferred to Axygen microtubes (Corning, Tewsbury, USA), and stored at −80°C. After approval of the Institutional Review Board, 15 frozen blood specimens stored for approximately 5 years were randomly selected.

Frozen blood specimens were thawed on ice and RNA extracted using three different kits (TRI reagent, PAXgene and NucleoSpin) according to the respective manufacturer protocols.

For TRI reagent extraction, 200 μl of each blood sample was incubated with 800 μl TRI reagent for 5 min at room temperature, followed by supplementation with 200 μl chloroform. After vigorous mixing and centrifugation at 12,000 g for 15 min at 4°C, the upper layer was transferred to a new tube. An aliquot of 500 μl isopropanol was added and the resulting mixture incubated for 10 min at room temperature, followed by centrifugation at 12,000 g for 15 min at 4°C to pellet RNA. The pellet was washed with 1 ml 75% ethanol and air-dried for 5 min. RNA was dissolved in 40 μl RNase-free water.

For PAXgene extraction, 300 μl of each blood sample was dispensed into 830 μl PAXgene reagent at a blood:reagent ratio of 2.5:6.9, which was the same as in the PAXgene Blood RNA Tubes (Beekman et al. 2009; Carrol et al. 2007; Krawiec et al. 2009). The mixture was incubated for 16 h at room temperature. After washing with 500 μl RNase-free water, the pellet was dissolved in 350 μl resuspension buffer and incubated with 300 μl binding buffer and 40 μl proteinase K for 10 min at 55°C in a shaker-incubator. The lysate was transferred into a PAXgene shredder spin column and centrifuged (at 18,000 g for 3 min). The flow-through fraction was mixed with 350 μl ethanol and transferred to a PAXgene RNA spin column. After washing the column with washing buffer 1, samples were incubated with 10 μl of DNase I for 15 min. PAXgene RNA spin columns were washed with washing buffer and RNA eluted with 40 μl elution buffer.

For NucleoSpin extraction, 200 μl of each blood sample was transferred to a collection tube to which 200 μl lysis buffer DL and 5 μl Proteinase K were added. The mixture was incubated for 15 min with vigorous shaking. Lysates were mixed with 200 μl of 70% ethanol, transferred to a NucleoSpin RNA Blood column, and washed with 350 μl MDB. Subsequently, 95 μl of rDNase was added to the column and incubated at room temperature for 15 min. The column was washed with Buffers RB2 and RB3, and RNA eluted using 40 μl RNase-free water.

### RNA assessment (yield, purity and integrity)

The RNA yield was estimated by measuring absorbance at 260 nm in a NanoDrop 2000 spectrophotometer (Thermo Fisher Scientific, Wilmington, DE, USA). RNA purity was calculated from the ratio of absorbance at 260 nm and 280 nm, and integrity assessed using the Eukaryote Total RNA Nano assay on the Agilent 2100 Bioanalyzer (Agilent Technologies, Santa Clara, CA, USA). The RNA integrity number (RIN) was calculated using the Agilent 2100 Bioanalyzer and accompanying software.

### RT-PCR analysis

cDNA was reverse-transcribed from total RNA using SuperScript II RNase transcriptase (Invitrogen, Carlsbad, CA, USA) following the manufacturer’s guidelines. In total, 200 ng of total RNA was used as template. The reaction mixtures were incubated in a 2720 Thermal Cycler (Applied Biosystems, Foster City, CA, USA). TaqMan Gene Expression Master Mix was used to perform real-time PCR (Applied Biosystems, Foster City, CA, USA). *18S rRNA* (Hs99999901_s1), *β-actin* (ACTB, Hs99999903_m1), *RPLP0* (Hs99999902_m1) and *HPRT1* (Hs99999909_m1) genes were amplified and quantified. Each reaction mixture (20 μl) was transferred to a 96-well plate, which was loaded onto the 7500 Real Time PCR System (Applied Biosystems, Foster City, CA, USA). Plates were heated to 50°C for 2 min and 95°C for 10 min, and subjected to cycles of 95°C for 15 sec and 60°C for 1 min. Data were expressed as cycle threshold (Ct) values.

### Statistical analysis

Experimental data are expressed as mean values and standard deviation. The performance of the three RNA extraction kits was compared with the Student’s *t*-test. Asterisks (***p < 0.001) represent statistically significant differences.

## References

[CR1] Barnes MG, Grom AA, Griffin TA, Colbert RA, Thompson SD (2010). Gene expression profiles from peripheral blood mononuclear cells Are sensitive to short processing delays. Biopresery Biobank.

[CR2] Beekman JM, Reischl J, Henderson D, Bauer D, Ternes R, Peña C, Lathia C, Heubach JF (2009). Recovery of microarray-quality RNA from frozen EDTA blood samples. J Pharmacol Toxicol Methods.

[CR3] Carrol ED, Salway F, Pepper SD, Saunders E, Mankhambo LA, Ollier WE, Hart CA, Day P (2007). Successful downstream application of the Paxgene Blood RNA system from small blood samples in paediatric patients for quantitative PCR analysis. BMC Immunol.

[CR4] Chai V, Vassilakos A, Lee Y, Wright JA, Young AH (2005). Optimization of the PAXgene blood RNA extraction system for gene expression analysis of clinical samples. J Clin Lab Anal.

[CR5] Elliott P, Peakman TC (2008). The UK Biobank sample handling and storage protocol for the collection, processing and archiving of human blood and urine. Int J Epidemiol.

[CR6] Fleige S, Pfaffl MW (2006). RNA integrity and the effect on the real-time qRT-PCR performance. Mol Aspects Med.

[CR7] Fleige S, Walf V, Huch S, Prgomet C, Sehm J, Pfaffl MW (2006). Comparison of relative mRNA quantification models and the impact of RNA integrity in quantitative real-time RT-PCR. Biotechnol Lett.

[CR8] Hebels DG, Georgiadis P, Keun HC, Athersuch TJ, Vineis P, Vermeulen R, Portengen L, Bergdahl IA, Hallmans G, Palli D, Bendinelli B, Krogh V, Tumino R, Sacerdote C, Panico S, Kleinjans JC, de Kok TM, Smith MT, Kyrtopoulos SA (2013). Performance in omics analyses of blood samples in long-term storage: opportunities for the exploitation of existing biobanks in environmental health research. Environ Health Perspect.

[CR9] Jackson DH, Banks RE (2010). Banking of clinical samples for proteomic biomarker studies: a consideration of logistical issues with a focus on pre-analytical variation. Proteomics Clin Appl.

[CR10] Jakovljevic KV, Spaxic MR, Malisic EJ, Dobricic JD, Krivokuca AM, Jankovic RN (2010). Comparison of phenol-based and alternative RNA isolation methods for gene expression analyses. J Serb Chem Soc.

[CR11] Jiang Z, Uboh CE, Chen J, Soma LR (2013). Isolation of RNA from equine peripheral blood cells: comparison of methods. Springerplus.

[CR12] Kang JE, Hwang SH, Lee JH, Park do Y, Kim HH (2011). Effects of RBC removal and TRIzol of peripheral blood samples on RNA stability. Clin Chim Acta.

[CR13] Krawiec JA, Chen H, Alom-Ruiz S, Jaye M (2009). Modified PAXgene method allows for isolation of high-integrity total RNA from microlitre volumes of mouse whole blood. Lab Anim.

[CR14] Pazzagli M, Malentacchi F, Simi L, Orlando C, Wyrich R, Günther K, Hartmann CC, Verderio P, Pizzamiglio S, Ciniselli CM, Tichopad A, Kubista M, Gelmini S (2013). SPIDIA-RNA: first external quality assessment for the pre-analytical phase of blood samples used for RNA based analyses. Methods.

[CR15] Rainen L, Oelmueller U, Jurgensen S, Wyrich R, Ballas C, Schram J, Herdman C, Bankaitis-Davis D, Nicholls N, Trollinger D, Tryon V (2002). Stabilization of mRNA expression in whole blood samples. Clin Chem.

[CR16] Thach DC, Lin B, Walter E, Kruzelock R, Rowley RK, Tibbetts C, Stenger DA (2003). Assessment of two methods for handling blood in collection tubes with RNA stabilizing agent for surveillance of gene expression profiles with high density microarrays. J Immunol Methods.

[CR17] Thörn I, Olsson-Strömberg U, Ohlsen C, Jonsson AM, Klangby U, Simonsson B, Barbany G (2005). The impact of RNA stabilization on minimal residual disease assessment in chronic myeloid leukemia. Haematologica.

